# Congenital Athymia: Genetic Etiologies, Clinical Manifestations, Diagnosis, and Treatment

**DOI:** 10.1007/s10875-021-01059-7

**Published:** 2021-05-13

**Authors:** Cathleen Collins, Emily Sharpe, Abigail Silber, Sarah Kulke, Elena W. Y. Hsieh

**Affiliations:** 1 Department of Pediatrics, Division of Allergy Immunology, Rady Children’s Hospital, University of California San Diego, San Diego, CA USA; 2 Trinity Life Sciences, Waltham, MA USA; 3 Enzyvant Therapeutics, Inc, Cambridge, MA USA; 4 Department of Pediatrics, Section of Allergy and Immunology, Children’s Hospital Colorado, University of Colorado School of Medicine, Aurora, CO USA; 5 Department of Immunology and Microbiology, University of Colorado School of Medicine, Aurora, CO USA

**Keywords:** Congenital athymia, midline defects, DiGeorge, T cells, immunodeficiency

## Abstract

Congenital athymia is an ultra-rare disease characterized by the absence of a functioning thymus. It is associated with several genetic and syndromic disorders including FOXN1 deficiency, 22q11.2 deletion, CHARGE Syndrome (Coloboma, Heart defects, Atresia of the nasal choanae, Retardation of growth and development, Genitourinary anomalies, and Ear anomalies), and Complete DiGeorge Syndrome. Congenital athymia can result from defects in genes that impact thymic organ development such as *FOXN1* and *PAX1* or from genes that are involved in development of the entire midline region, such as *TBX1* within the 22q11.2 region, *CHD7*, and *FOXI3*. Patients with congenital athymia have profound immunodeficiency, increased susceptibility to infections, and frequently, autologous graft-versus-host disease (GVHD). Athymic patients often present with absent T cells but normal numbers of B cells and Natural Killer cells (T^−^B^+^NK^+^), similar to a phenotype of severe combined immunodeficiency (SCID); these patients may require additional steps to confirm the diagnosis if no known genetic cause of athymia is identified. However, distinguishing athymia from SCID is crucial, as treatments differ for these conditions. Cultured thymus tissue is being investigated as a treatment for congenital athymia. Here, we review what is known about the epidemiology, underlying etiologies, clinical manifestations, and treatments for congenital athymia.

## Introduction

Congenital athymia is an ultra-rare condition [1] characterized by the absence of a thymus at birth. The thymus is crucial for the maturation and selection of T cells, and infants born without a thymus suffer from profound immunodeficiency [2]. Failure to promptly diagnose this disease and institute measures to prevent exposure to infectious agents can be fatal. Currently, all 50 states in the USA offer newborn screening for T cell receptor excision circles (TRECs) which will identify infants who may have congenital athymia in addition to severe combined immunodeficiency (SCID) [3, 4]. The diagnosis requires confirmation of low naïve T cells by flow cytometry [5].

Multiple genetic abnormalities, congenital syndromes, and environmental factors are associated with congenital athymia, and the care of these infants is complex. While 22q11.2 deletion-associated with DiGeorge Syndrome (DGS) is the most commonly described genetic defect associated with congenital athymia, *FOXN1*, *PAX1,* and others have also been identified as potentially causative [5–8]. Among patients with DGS, the majority have partial DGS (pDGS), which is characterized by T cell deficiency, but not athymia [9–11]. Complete DGS (cDGS) refers to patients with congenital athymia; cDGS patients account for a minor proportion of all DGS patients [2, 11, 12]. CHARGE Syndrome (Coloboma, Heart defects, Atresia of the nasal choanae, Retardation of growth and development, Genitourinary anomalies, and Ear anomalies) has also been shown to be associated with congenital athymia [5]. Environmental exposures have also been implicated, such as diabetic embryopathy and retinoic acid exposure [5, 13].

Hallmarks of congenital athymia include a profound T cell deficiency, frequent infections, susceptibility to opportunistic infections, and propensity to develop autologous graft-versus-host disease (GVHD), or in the clinical picture of cDGS, as having an ‘atypical’ phenotype [5, 7]. Autologous GVHD is often the term used to refer to auto-reactive T cells that escaped T cell selection due to lack of thymus. These T cells often produce a cellular infiltrate and organ damage [14, 15]. It is critical to ensure that these infants do not receive immunizations prior to immune reconstitution as live vaccines may be fatal. Hematopoietic stem cell transplantation has not proved effective in treating these patients [16, 17]. A therapy currently under investigation is the implantation of cultured thymus tissue [14, 18].

## Etiology of Congenital Athymia

The thymus is a crucial element of the immune system because it is the only organ where thymocytes can mature, be selected, and ultimately survive to become naïve T cells [19]. T cell precursors emerge from the bone marrow and migrate to the thymus for maturation. These cells enter the thymus at the junction of the cortex and medulla and travel to the subcapsular region of the thymic cortex. In the subcapsular region, they undergo differentiation that results in expression of the T cell receptor (TCR). They travel from the subcapsular region to the cortex where they begin to express CD4 and CD8 receptors. In the cortex, T cell precursors undergo positive selection via interactions with cortical thymic epithelial cells (cTECs) expressing self-antigens on MHC I and II. T cell precursors then travel to the medulla and undergo negative selection through interaction with medullary thymic epithelial cells (mTECs) that express tissue restricted antigens from different organs through the transcriptional regulator Aire and others [20, 21]. Subsequent downregulation of either CD4 or CD8 produces naïve single positive cells that are ready to exit the thymus and enter the peripheral bloodstream.

### Thymus Development Overview

**Fig. 1:**
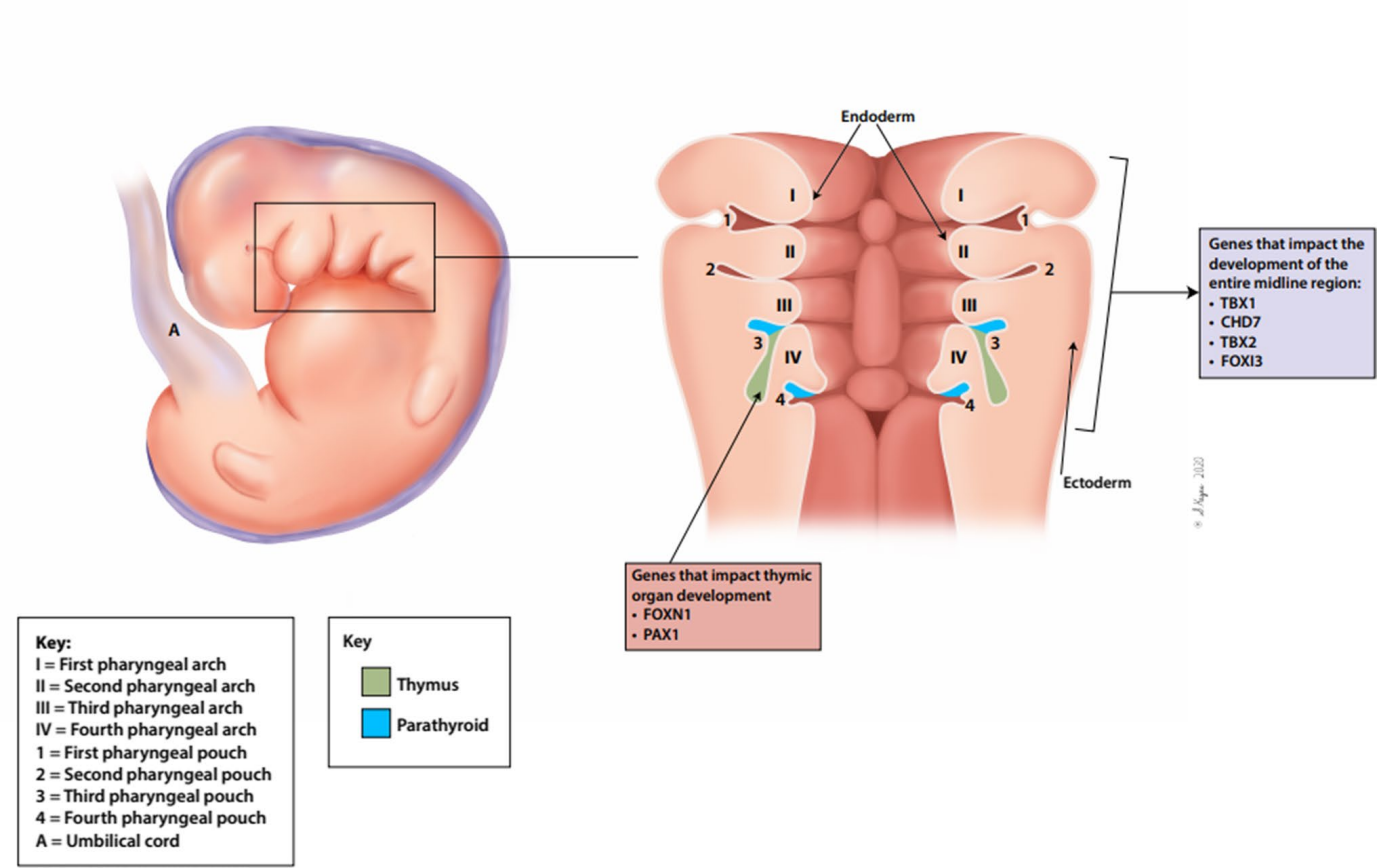
Genetic etiologies of congenital athymia and impact on embryogenesis. Artistic rendering of the different etiologies associated with congenital athymia and how they impact the developing embryo. Genetic etiologies can be categorized by whether the impacted gene is involved in development of the entire midline region or more directly in thymic organ development

**Fig. 2:**
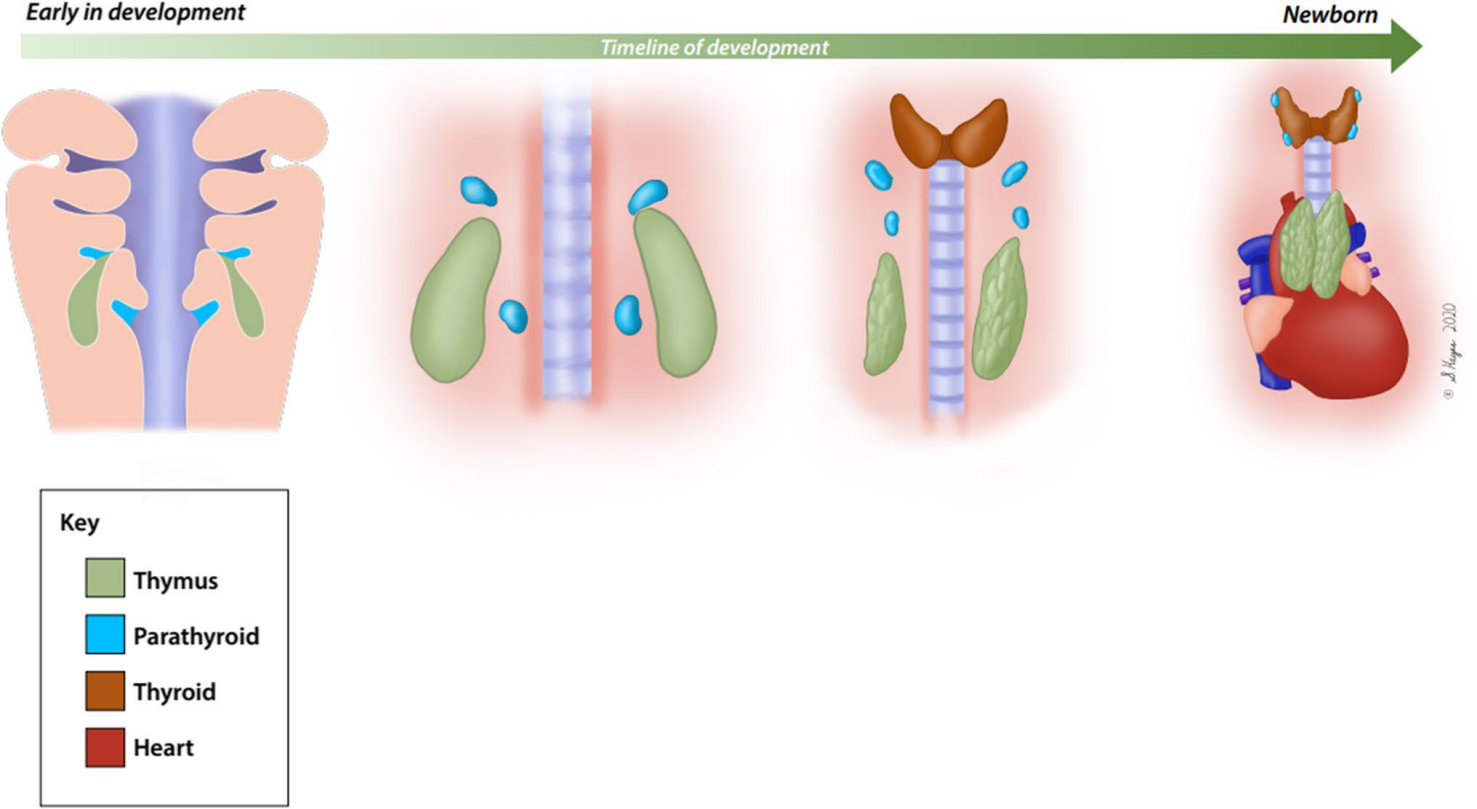
Normal thymus embryogenesis. Artistic rendering of the development of the thymus and parathyroid from the third pharyngeal pouch. Spatial and functional separation occurs early, with the thymus in the ventral posterior region and the parathyroid more anterior. During development, the thymus migrates caudally and medially to its final position in the anterior part of the thorax and fuses with the developing thymus from the contralateral side

One of the most characteristic features of human embryonic development of the head and neck are the pharyngeal arches (Fig. 1). The pharyngeal arches are lined internally by endoderm, externally by ectoderm, and each contains a mesenchymal tissue center derived from mesoderm and neural crest cells. Pharyngeal pouches develop from the internal endoderm and give rise to different organs. During embryonic development, the third pharyngeal pouch endoderm gives rise to the thymus and parathyroid glands, as shown in Fig. 2. Several genes have been identified that regulate the early phases of pharyngeal pouch formation and patterning, as well as subsequent thymus organ development (Table 1).

**Table 1:** Genes implicated in abnormal development of the thymus

Gene	Role in thymus development	Associated conditions	Non-immune manifestations of associated conditions
*FOXN1*	Development, differentiation, and maintenance of thymic epithelial cells (TECs) in embryonic and postnatal life [22–24, 26]	FOXN1 deficiency (Nude/SCID) [27]	Congenital alopecia and nail dystrophy
*PAX1*	Early expression in the pharyngeal pouches and TECs [35] Possible role in establishing milieu for T cell maturation	Otofaciocervical Syndrome Type 2 [6, 36]	Facial dysmorphism, external ear anomalies, hearing loss, branchial cysts or fistulas, shoulder girdle anomalies, and mild intellectual disability
*TBX1*	Pharyngeal arch artery formation and pharyngeal segmentation Possible role in establishing parathyroid fate [41–45]	22q11.2 Deletion Syndrome DiGeorge Syndrome [8]	22q11.2 Deletion Syndrome: Developmental delay, ear anomalies and hearing loss, velopharyngeal insufficiency, and cleft lip and/or palate DiGeorge Syndrome: Congenital heart defects, hypoparathyroidism
*CHD7*	Pharyngeal arch artery and pouch formation and TEC development [47, 48]	CHARGE Syndrome [51]	Eye coloboma, heart defects, choanal atresia, retardation of growth and/or development, genital abnormalities, and ear abnormalities and/or deafness
*FOXI3*	Pharyngeal segmentation [105]	N/A	N/A
*TBX2*	Role not well defined	N/A	N/A

Congenital athymia can result from genetic defects that are (1) specific to thymic organ development or (2) involved in the broader development of the entire midline region (Fig. 1).

### Genetic Defects Specific to Thymic Organ Development

#### FOXN1

Forkhead Box N1 *(FOXN1)* is the most well-known gene specific to thymic development. *FOXN1* belongs to the forkhead box gene family of transcription factors and is involved in the development, differentiation, and maintenance of TECs in embryonic and postnatal life, and growth and differentiation of skin epithelial cells [22–26]. The clinical presentation of patients with homozygous mutations in *FOXN1* illustrates its key role. Patients characteristically present with congenital athymia, congenital alopecia, and nail dystrophy [27, 28]. Patients with homozygous *FOXN1* mutations typically have low T cell numbers and function, and normal B and Natural Killer (NK) cells, though possible reduced B cell function. To date, three distinct homozygous mutations have been identified in approximately 10 patients [7, 28–31]. All three mutations result in loss of function of the protein; two are located in the N-terminus domain while one is located in the forkhead domain.

Heterozygous mutations in *FOXN1* have also been described [29, 32–34]. Bosticardo et al. identified 20 distinct heterozygous loss-of-function *FONX1* gene variants in pediatric and adult patients [33]. Variants occurred throughout the *FOXN1* gene, but clustered in the forkhead and C-terminal domains. Most pediatric patients with heterozygous *FOXN1* mutations did not have complete congenital athymia but instead presented with low levels of TRECs (identified by newborn screening) and T cell lymphopenia at birth. Importantly, their lymphopenia tended to improve over time; longitudinal analysis found that although CD8 + T cell lymphopenia persisted, CD4^+^ T cell lymphopenia became less severe past 2 years of age. For adult patients, T cell counts were typically within normal range, with the exception of CD8^+^ T cell counts which were lower than normal. Though difficult to interpret the significance of these results, a thymic shadow was absent in 4 out of 13 pediatric patients evaluated with a heterozygous *FOXN1* variant. To understand the underlying cause for this immune phenotype, the authors created *Foxn1*^*nu/*+^ mice and found a considerable decrease in early thymic progenitors and expression of *Foxn1* transcriptional targets early in the first weeks of life that trended toward normal over time. Consistent with the nude/SCID phenotype, several pediatric and adult patients with heterozygous *FOXN1* mutations also had nail dystrophy, slight hair thinning or hair loss, and eczema or other atopic dermatitis manifestations [33].

Compound heterozygous mutations have also been reported in two patients described as having a presentation consistent with T^−/lo^B^+^NK^+^ SCID, but without defects in hair and nails [34]. Both patients had low or absent naïve T cells. One of the patients had multiple infections and died of parainfluenza virus prior to one year of life. Separate compound heterozygous *FOXN1* mutations were identified in the two patients [34].

#### PAX1

Paired Box 1 *(PAX1)* is a member of the paired box family of transcription factors that drive differentiation of tissues. In mice, expression of *Pax1* can be detected early in the pharyngeal pouch endoderm, including the epithelium of the third pharyngeal pouch [35]. During thymus organogenesis, a large proportion of precursor and TECs express *Pax1*, however, this expression decreases over time, and adult mice have few cortical epithelial cells with significant *Pax1* expression [35]. These data support the hypothesis that *Pax1* expression in thymic epithelium plays a role in establishing the milieu for T cell maturation [35].

Several papers have described patients with mutations in *PAX1* and autosomal recessive otofaciocervical syndrome type 2 (OTFCS2). Yamazaki et al. identified biallelic, loss-of-function *PAX1* mutations in six patients with OTFCS2 they described as linked to a syndromic form of SCID due to altered thymus development; two included patients were also previously described by Paganini et al. [36, 37]. Patients typically had marked T cell lymphopenia. While the authors refer to these patients’ immune dysfunction as SCID, it is likely congenital athymia given that the patients failed to develop T cells following successful engraftment of bone marrow transplantation and several had documented absence of a thymic shadow on chest x-ray [36, 37]. Three separate biallelic *PAX1* mutations were identified [37].

Patil et al. also described two siblings who presented with global developmental delay, hearing impairment, external ear malformations, and facial dysmorphism associated with OTFCS2. Exome sequencing revealed a homozygous *PAX1* mutation [6]. Both patients had a documented absent thymic shadow, but no further immune details were provided.

### Genetic Defects that Impact Development of the Entire Midline Region

The most common genetic syndromes associated with defects in thymus development are 22q11.2 deletion syndrome and CHARGE syndrome. The genes implicated in these respective disorders, *TBX1* and *CHD7*, play a role in development of the entire midline region, and as a result, patients with these syndromes present with a constellation of symptoms.

### 22q11.2 Deletion and TBX1

Among the genes that impact midline development generally, the most well-known association with immune deficiency is T-Box Transcription Factor 1 (*TBX1*). *TBX1* is part of the approximately 30 genes in the 22q11.2 region that are commonly deleted in DGS [38–40]. *TBX1* belongs to the T-box family of transcription factors, a highly conserved group regulating vertebrate limb and heart development. The downstream targets of *TBX1* are not well understood. Starting early in embryogenesis in mice, *Tbx1* is expressed in the pharyngeal structure, including the endoderm and arch mesenchyme, and is thought to play a role in pharyngeal arch artery formation and pharyngeal segmentation [41–43]. Later in embryogenesis, expression of *Tbx1* in the third pharyngeal pouch becomes more restricted to the parathyroid domain [41, 44]. In fact, *Tbx1* expression may need to stop in order for TEC proliferation and differentiation to occur [45].

In humans, *TBX1* mutations and *TBX1* haploinsufficiency are thought to be the principal cause for the congenital defects associated with 22q11.2 deletion and DGS. A review of cDGS patients found that over 50% had 22q11.2 hemizygosity [5]. Most patients with 22q11.2 deletion syndrome have a 1.5–3 Mb deletion. Despite this, *TBX1* is the only gene that has been discovered to date to demonstrate a direct relationship between haploinsufficiency and replication of the 22q11.2 deletion syndrome and DGS phenotype [8]. Moreover, several mutations in *TBX1*’s T-box binding domain have been shown in patients with the characteristic 22q11.2 deletion syndrome and DGS phenotype but lacking a 22q11.2 deletion [8]. Two mutations were identified in patients with conotruncal anomaly face syndrome/velocardiofacial syndrome, and one mutation in a patient with DGS and absent thymus; no further immune evaluations were performed.

In the mouse, *Tbx1* has been shown to regulate proper development of the pharyngeal arch arteries in a gene dosage-dependent manner [38]. *Tbx1*^±^ embryos have defects of the fourth pharyngeal arch arteries, whereas *Tbx1*^−/−^ embryos have developmental defects of the second, third, fourth, and sixth pharyngeal arches and arch arteries and the second, third, and fourth pharyngeal pouches [38, 39, 41]. Moreover, *Tbx1*^±^ embryos do not demonstrate significant thymus abnormalities, whereas thymic aplasia is a characteristic feature of *Tbx1*^−/−^ embryos [39, 46]. Xu et al. demonstrated that the impact of *Tbx1* mutation on proper development of the mouse thymus also varies by the time point of its deletion. If *Tbx1* is deleted prior to E9.5 in development, then the thymus is absent. Whereas, if *Tbx1* is deleted at E9.5 or E10.5, a hypoplastic thymus forms, and if *Tbx1* is deleted after E11.5, there is no impact on thymus morphogenesis [42].

#### CHD7

Chromodomain Helicase DNA Binding Protein 7 (*CHD7*) is an ATP-dependent nucleosome remodeling factor. Randall et al. found that *Chd7*^±^mouse embryos had defects in artery development of the fourth pharyngeal arch [47]. Approximately 11% of heterozygous *Chd7*^±^ embryos had a small or ectopically placed thymus at E14.5; this thymus hypoplasia correlated with downregulation of *Foxn1* [47]. In zebrafish, *Chd7* has also been shown to play a role in TEC development from the pharyngeal endoderm and through downstream regulation of *Foxn1* [48].

*CHD7* mutations have been implicated in CHARGE syndrome; approximately 60–65% of patients with CHARGE have a mutation in *CHD7* [49, 50]. Wong et al. reviewed the immunological aspects of CHARGE across multiple studies and found that thymic aplasia has been reported in 27 of 59 patients with CHARGE and in 16 of 36 patients with a proven variant in *CHD7*. However, T cell evaluations were not consistently performed, and in some cases, thymic aplasia was documented only via imaging or surgery [51]. When patients with thymic aplasia did undergo immune evaluations, they typically demonstrated marked T cell lymphopenia, including reports of low naïve T cells, reduced T cell function, and normal B and NK cell numbers [52–57]. In addition, several patients without documented thymic aplasia or with severe hypoplasia had low naïve T cells or reported absence of T cells [58–60]. CHARGE has also been reported in patients with congenital athymia without genetic sequencing to confirm mutations in *CHD7*. Markert et al. reported association of cDGS and CHARGE in 14 of 54 patients undergoing evaluation for cultured thymus tissue implantation [5].

#### TBX2 and FOXI3

Other possible genes implicated in proper thymus development include *TBX2*, and *FOXI3*. A recent study found that four individuals with clinical findings reminiscent of DGS, were heterozygous for *TBX2* variants; one family of three with the same genetic defect and a separate individual [61]. In the first family, all three members had abnormally low T cell numbers, and one required investigational cultured thymus tissue implantation. The fourth subject was from another family and did not have significant immune deficiency. Additionally, *FOXI3* was recently implicated in a 22p11.2 microdeletion in several patients with features resembling DGS. These patients had T cell lymphopenia identified by low TRECs through newborn SCID screening and an inverted kappa/lambda ratio on flow cytometric analysis [62]*.*

### Environmental Etiologies

Several environmental etiologies are associated with congenital athymia. Diabetic embryopathy is associated with altered fetal thymus size, and other congenital abnormalities such as renal agenesis and butterfly vertebrae [63, 64]. Thymic aplasia has been demonstrated in infants of diabetic mothers, and Markert et al. found that 15% of patients undergoing evaluation for cultured thymus tissue implantation were born to mothers who had diabetes [5, 64]. The underlying mechanism is not well understood.

Fetal exposure to retinoic acid is also associated with a DGS phenotype, including thymus developmental abnormalities reported as thymic aplasia and ectopia, and hypoplasia [13, 65, 66]. A possible mechanism for the abnormalities in thymus development is alteration of *Tbx1* and/or *Pax1* expression by retinoic acid exposure [67, 68].

## Epidemiology of Congenital Athymia

Congenital athymia is reported in the literature almost exclusively as a clinical feature of syndromic and genetic conditions. Since these reports often describe athymia associated with specific conditions, its overall incidence across all etiologies is not well understood. 22q11.2 deletion syndrome and CHARGE syndrome are the two most common genetic defects associated with thymic defects. The incidence of 22q11.2 deletion is estimated at 1:4000–1:9700 live births, and the incidence of CHARGE is estimated at 1:8500 live births [69–72]. A subset of patients within these genetic disorders present with immunodeficiency, congenital heart defects, and hypoparathyroidism; they are described as having DGS. As mentioned in the foregoing, pDGS refers to patients with T cell deficiency but not athymia, whereas cDGS refers to patients with congenital athymia.

In published studies, congenital athymia is most commonly reported in the context of cDGS, with or without 22q11.2 genetic deletion [5, 73]. Recent widespread adoption of newborn screening for SCID in the USA has played a valuable role in helping to establish the incidence of cDGS; patients with congenital athymia are identified by a positive finding on a SCID screen due to absence of TRECs. Kwan et al. found that across 11 statewide newborn SCID screening programs, cDGS was diagnosed in 1:1,010,027 births [4]. Consistent with that estimate, the newborn SCID screening program in California (2010–2017) found that out of 3,252,156 reported births, cDGS was diagnosed in 1:813,039 births [74]. These low estimates underscore the advances in newborn screening in detecting congenital athymia.

In addition to cDGS, congenital athymia is also a hallmark of FOXN1 deficiency. Despite this, no precise incidence is known, and only approximately 10 cases of homozygous FOXN1 deficiency have been reported in the literature to date [7, 28–31]. Other underlying etiologies of congenital athymia do not have a well-established incidence in the literature.

## Diagnosis of Congenital Athymia

**Fig. 3:**
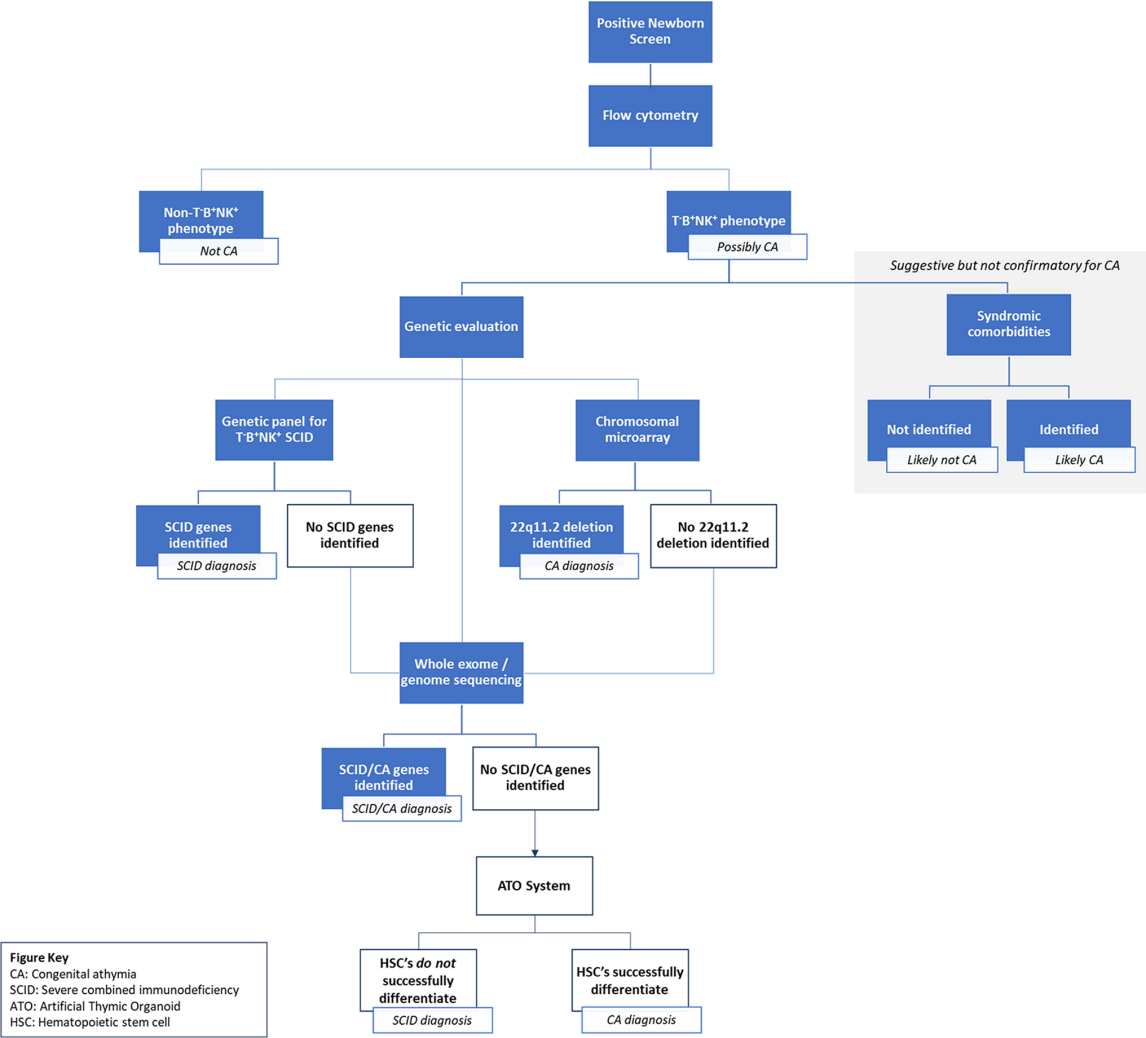
Congenital athymia diagnostic pathway. Schematic of the diagnostic pathway for congenital athymia from initial identification through newborn screening to final diagnosis, including steps for how to accurately differentiate athymia from T^−^B^+^NK^+^ SCID

Congenital athymia is often first identified through newborn screening for SCID (Fig. 3), which is required in all 50 states as of 2018 [3, 4, 74]. SCID screening evaluates immune function via quantification of TRECs by polymerase chain reaction using DNA isolated from dried blood spots [3, 74]. TRECs are episomal DNA excision products formed during T cell receptor rearrangement in the thymus [75]. Low or undetectable TRECs are considered a positive finding during SCID screening; reported cutoffs for a positive finding vary by state, ranging from fewer than 4 to 252 copies per microliter (µL) [4, 74]. Patients with congenital athymia fall within the positive group.

All positive patients subsequently undergo complete and differential blood counts and lymphocyte phenotyping by flow cytometry [4, 62, 74]. Congenital athymia patients are identified by their profoundly low naïve T cells. Patients with congenital athymia will have less than 50 naïve T cells per cubic millimeter (mm^3^) or naïve T cells comprising less than 5% of the total T cells [5, 14]. Since patients with congenital athymia lack T cells but have normal numbers of B cells and NK cells, they present with a T^−^B^+^NK^+^ phenotype. Complicating this, a subset of SCID patients also present with a T^−^B^+^NK^+^ phenotype [76]. The initial approach to differentiate these patients is through known mutations in genes that cause athymia (described in the foregoing), or T^−^B^+^NK^+^ SCID, such as *IL-7R* and *CD3D* [76]. A genetic panel for known T^−^B^+^NK^+^ SCID gene mutations, whole exome or genome sequencing, and chromosomal microarray can be used to help differentiate these patients. If there are no SCID-causing genetic defects identified, and chromosomal microarray is not enlightening for syndromic defects, then proper diagnosis is challenging. Accurate identification of the underlying cause for immunodeficiency in these patients is critical for informed treatment decisions, i.e., receipt of hematopoietic stem cell transplant versus cultured thymus tissue implantation. Seet et al. recently developed an artificial thymic organoid (ATO) system that supports in vitro differentiation, positive selection, and maturation of human T cells from cord blood, bone marrow, and/or peripheral blood CD34^+^ hematopoietic stem cells, dependent on the collaborating organization [77–79]. This system can evaluate the ability of hematopoietic cells to become mature T cells, such that if the isolated cells are able to differentiate appropriately, a diagnosis of SCID can be excluded.

Patients can also be evaluated for comorbidities that may reveal athymia as part of a clinical picture of a syndromic condition, such as cDGS or CHARGE. While a positive finding of a syndromic comorbidity may indicate congenital athymia versus SCID, it should not be used to establish a definitive diagnosis without an additional confirmatory method. Of note, although confirmation of congenital athymia has been reported in the literature through imaging studies (i.e. chest x-ray [33]), complete absence of the thymus must be confirmed in patients based on laboratory findings. Imaging can fail to determine the presence of a thymus in the mediastinum as it may be small and easily missed, or it may not have descended properly [80].

Subsequent testing in patients with suspected congenital athymia may include a mitogen T cell proliferation assay via flow cytometry, which is available at specialized immune diagnostic laboratories and requires only a small blood volume. Mitogen T cell proliferation assay is frequently sent as part of the initial diagnosis of T cell lymphopenia; these tests use phytohemagglutinin or concanavalin A to stimulate T cell responses independently of antigen specificity. In general, patients with congenital athymia have low mitogen stimulation, typically less than 20-fold proliferative response, like patients with SCID [2, 81]. However, in the face of oligoclonal T cell expansion and autologous GVHD, patients with congenital athymia may demonstrate responses to mitogens [15].

Some patients with congenital athymia may develop high numbers of circulating T cells and can demonstrate a response to mitogens. These patients are uniquely characterized by a rash and associated lymphadenopathy [15, 30, 82, 83]. Biopsy of the rash reveals features of an inflammatory response in the stratum corneum, epidermis, and dermis, including infiltrating T cells, exocytosis, parakeratosis, and spongiosis [83]. This phenotype develops at some point after birth [18]. The last published breakdown between typical and atypical phenotype was in 2009 and noted that 30% of patients had an atypical phenotype [14]. We expect that the percent of atypical patients has increased overtime due to widespread newborn screening and the institution of supportive care shortly after birth. These advances in early diagnosis and isolation have allowed patients to live longer, likely increasing the proportion of patients transitioning to the atypical phenotype [3]. The high numbers of circulating T cells in these patients are oligoclonal (clonal expansions of T cells expressing similar T cell receptor variable regions) and lack expression of naïve T cell markers (including, CD45RA, CD62L, and CD31) [15]. These T cells have been shown to develop through extrathymic proliferation and display a predominantly memory phenotype, expressing T cell marker CD45RO [30, 37, 84–86]. A finding of significant CD45RO^+^ T cells by flow cytometry in athymic infants is an indicator for this atypical phenotype. Possible further evaluation of T cells in these patients may include T cell receptor β chain variable repertoire analysis by flow cytometry or spectratyping to establish their oligoclonality [15]. Additionally, although these T cells may functionally proliferate after stimulation with mitogens, they do not respond to antigens and therefore are not protective against infection [15]. Notably, the presenting atypical phenotype can appear similar to Omenn syndrome secondary to SCID and maternal engraftment [87, 88]. Chimerism studies can be used to distinguish maternal engraftment versus Omenn syndrome, whereas autologous GVHD and Omenn syndrome appear immunologically the same.

Patients with suspected or confirmed congenital athymia should undergo testing to identify possible associated genetic and syndromic conditions. Genetic testing and chromosomal microarray can help to identify conditions such as 22q11.2 deletion syndrome (22q11.2 hemizygosity), CHARGE (*CHD7* mutations), or FOXN1 deficiency (*FOXN1* mutations). Patients should also be evaluated for clinical manifestations of associated conditions (described in Table 1).

## Clinical Manifestations of Congenital Athymia

The clinical manifestations of congenital athymia are a direct result of the absence of the thymus and the inability to produce immunocompetent T cells. Two fundamental categories describe the clinical features of the disease: profound T cell immunodeficiency and autologous GVHD. As congenital athymia is often a feature of broader syndromic or genetic conditions, patients can present with a constellation of symptoms.

### Infections

Prior to widespread availability of newborn screening, patients with congenital athymia presented in the first few months of life with recurrent and persistent infections, often categorized as severe [2, 16, 51, 82, 89]. T cell immunodeficiency leads to an increased susceptibility to bacterial, viral, and fungal infections. Pneumonias occur at a particularly high rate in these patients [16, 82, 89]. One multicenter survey of patients with congenital athymia found that ~ 30% of patients developed pneumonia [16]. These pneumonias were caused by *Pseudomonas* spp including *P. aeruginosa,* as well as *C. albicans*, *S. aureus*, *S. pneumoniae*, and Haemophilus parainfluenza. Pneumonias can be recurrent and severe and patients can develop chronic lung disease [16, 82, 89]. Other severe pulmonary infections have been reported with respiratory syncytial virus and *M. bovis* [7, 82, 89]. Gastrointestinal infections are also frequent among this population, including rotavirus, norovirus, enterovirus, *M. bovis*, and *C. difficile* infections [7, 16, 82, 89]. These infections can lead to failure to thrive, malabsorption, and diarrhea. While infections are most commonly reported in pulmonary and gastrointestinal organs, patients with congenital athymia can present with a myriad of other infection types. Infections of the urinary tract from *K. pnuemoniae*, *E. faecium*, and echovirus have been reported, as well as infections of the head, ears, nose, and throat including meningitis, sinusitis, mastoiditis, and thrush [16, 82, 89]. Of particular note, athymic patients also experience sepsis, which can be associated with mortality in this patient population [16, 81, 82, 89, 90].

The profound nature of the T cell immunodeficiency in patients who lack a thymus also puts them at significant risk from life-threatening opportunistic infections, including Cytomegalovirus (CMV), *Candida*, *Pneumocystis Carinii* and Human Herpesvirus 6 infections [7, 16, 81, 82, 89, 91]. CMV infection is of particular concern, leading clinicians to recommend that mothers of infants with primary immunodeficiencies not breastfeed to prevent potential CMV transmission to the infant [92–94]. CMV infection is an important consideration for eligibility for cultured thymus tissue implantation, as CMV infections have been reported to be fatal in these infants [18].

### Autologous GVHD

Potentially the most challenging aspect of managing patients with congenital athymia is their propensity to develop inflammatory autoimmune conditions, as described in the foregoing as autologous GVHD. In congenital athymia, T cells can undergo extrathymic oligoclonal expansion. These cells confer little to no protective immunity and can infiltrate organs causing autologous GVHD. Patients with oligoclonal T cell expansion typically have a characteristic eczematous rash and associated lymphadenopathy. Infiltrating T cells can cause transaminitis, and enteropathy in the gastrointestinal system [14]. Autologous GVHD contributes to increased morbidity in these patients, leading to higher susceptibility to infections. Treatment for these patients involves immunosuppression typically with steroids or calcineurin inhibitors, with accompanying potential adverse events such as hypertension, renal complications, and thrombocytopenia [14, 82].

### Autoimmunity

In addition to autologous GVHD, patients with congenital athymia have other manifestations of autoimmune mediated processes, such as hypothyroidism, autoimmune thyroiditis, and Coombs-positive hemolytic anemia [16, 89, 90]. These autoimmune processes have also been described in patients with 22q11.2 deletion syndrome without congenital athymia and include rheumatoid arthritis, idiopathic thrombocytopenia, hemolytic anemia, and thyroid disease [95].

## Treatment of Patients With Congenital Athymia

### Isolation

Management of patients with congenital athymia focuses on supportive care to reduce the risk of infection until the underlying immune deficiency can be corrected. Similar to other primary immunodeficiencies, as soon as congenital athymia is suspected after birth, it is recommended that neonates in the hospital be placed in reverse isolation with air filtering systems such as high-efficiency particulate air (HEPA) and positive pressure laminar air flow (LAF), though isolation protocols vary by hospital [27, 94, 96, 97]. All visitors are required to follow strict infectious disease prevention measures such as surgical hand washing protocols, hair covers, masks, shoe covers, sterile gowns and gloves [93, 94, 97]. If an athymic patient is discharged, isolation and hygiene procedures must also be maintained at home. This includes frequent handwashing, changing clothes/sanitizing upon re-entering the house, and restricting visitors in the home.

### Prophylaxis

In addition to isolation, patients with congenital athymia should begin antimicrobial prophylaxis to prevent bacterial, viral and fungal infections [18, 93, 94]. During this time, patients are closely monitored and treated for all infections.

### Immunoglobulin

Although patients with congenital athymia often have normal numbers of B cells, their B cell function is usually reduced. Therefore, patients should receive immunoglobulin replacement [5, 18, 94].

### Vaccination Avoidance

Recommendations for vaccine eligibility depend on the extent of the patient’s immunodeficiency [98]. Patients with partial T cell deficiency, such as pDGS, may be able to receive select live vaccines, based on their degree of immunodeficiency and T cell function; in general, retrospective studies have supported their safety in pDGS [99, 100]. For patients with complete T cell deficiency, such as congenital athymia associated with cDGS, all live vaccines are contraindicated [98]. Moreover, it is likely that all vaccines in athymic patients are ineffective prior to thymic implantation due to the importance of T cell help in directing appropriate antibody responses.

### Blood Products

All blood products given to the patient should be irradiated to prevent GVHD and tested and confirmed seronegative for CMV [5, 93, 96].

### Immunosuppression

Patients with oligoclonal T cells and/or elevated proliferative responsiveness to mitogens should also receive immunosuppression (such as steroids or calcineurin inhibitors) to manage their inflammatory reaction and reduce the risk of autologous GVHD [18, 82]. In addition, they may require anti-thymocyte globulin prior to receiving a cultured thymus tissue implantation.

### Syndromic Comorbidities

Though not directly related to their immunodeficiency, patients with congenital athymia also require management for defects due to associated syndromic conditions. Approximately 80% of patients with cDGS require calcium supplementation due to coexisting hypoparathyroidism that may manifest as newborn seizures [5]. This treatment needs to be monitored closely as severe hypocalcemia can lead to cardiac arrest and seizures, and supplementation can lead to nephrocalcinosis [2, 90]. Patients with congenital athymia as part of a cDGS clinical picture often require surgical care for heart defects [5]. cDGS patients may require tracheostomy for laryngomalacia, tracheomalacia, or ventilator-dependence secondary to cardiac problems [90]. Central lines for venous access and feeding tubes for nutritional supplementation are commonly required. [90]. Pediatric specialties involved in the care of these patients include neonatology, immunology, cardiology, endocrinology, nephrology, genetics, rheumatology, and hematology.

### Hematopoietic Stem Cell Transplant

Hematopoietic stem cell transplant (HSCT) has been performed in congenital athymia patients with relatively little success [16, 17]. Survival after HSCT in patients with congenital athymia is low compared to patients with SCID (41% compared to as high as 90%, respectively) [16, 101, 102]. Moreover, significant adverse events have been reported in athymic patients post-HSCT, including GVHD in ~ 50% of patients [16]. Immune reconstitution in athymic patients post-HSCT is also poor, with no clear evidence of successful regeneration of naïve T cells [16].

### Cultured Thymus Tissue Implantation

Establishing a functional thymic environment is essential to achieving full immune reconstitution in patients with congenital athymia. Cultured thymus tissue implantation (CTTI), historically described as thymus transplantation, has been shown in clinical trials to restore a functional T cell compartment via the migration of the recipient’s bone marrow derived stem cells to the implanted cultured thymus tissue, followed by subsequent development of immunocompetent naïve T cells [14, 18]. CTTI is currently being investigated in the USA by Dr. Louise Markert at Duke University [18] and has been performed in patients with cDGS, FOXN1 deficiency, and associated genetic and syndromic conditions including 22q11.2 hemizygosity, CHARGE, and diabetic embryopathy [5, 7]. The investigational use of cultured thymus tissue implantation has been previously described in detail [14, 18].

In the clinical trials of CTTI, patients were closely monitored post-implantation as full immune reconstitution took many months. Prior to reconstitution, patients were maintained in isolation and continued to receive immunoglobulin replacement and prophylactic antimicrobials. Criteria were developed to define the stepwise immune reconstitution and corresponding tapering of supportive care in these patients [18]. If patients were on immunosuppressive drugs prior to implantation, they were weaned from immunosuppression when naïve T cells were greater than 10% of the total T cell count [18]. Circulating naïve T cells were typically detected in patients approximately 6 months post-implantation, and peaked around 1–2 years post-implantation [14, 18]. Once patients were successfully weaned from immunosuppressive drugs, were 9 months post-implantation, had normal trough IgG levels for age, and demonstrated a proliferative response to mitogens of at least 100,000 counts per minute, immunoglobulin replacement therapy was discontinued [18]. Patients typically developed a normal proliferative response to mitogens 9–12 months post-implantation, while B cell function normalized 1–2 years post-implantation [14, 18]. Biopsies 2–3 months post-implantation demonstrated thymopoiesis in approximately 80% of patients, and all surviving patients with thymopoiesis on biopsy developed naïve T cells and adequate T cell function [103].

The efficacy and safety of CTTI was last reported in the peer reviewed literature in 2010 [18]. Since that time, there has been a significant expansion of the clinical trial program at Duke from 60 subjects reported in 2010 to 105 subjects in 2021 [104]. The results of this clinical trial update are not available at the time of this publication. The 2010 report noted that survival post-implantation was 72% across 60 patients [18]. Post-implant mortality most often occurred in patients prior to full immune reconstitution; 2 patients died more than one-year post-implant [18]. Survival curves show that the death rate diminishes 6–12 months post-implantation when immune reconstitution would be expected to be achieved [5, 14, 18]. Across the 60 patients reported in 2010, the majority of deaths were a result of infections, most commonly viral [18]. While infections were one of the most common adverse events post-implantation, autoimmune disorders were also reported. Thyroid disease has been reported in 13 patients following implantation [18]. As a result, patients undergo thyroid function evaluation periodically [18]. Cytopenias and other autoimmune conditions have also been reported post-implant [18]. It is unclear if autoimmune disease is a function of the underlying congenital athymia or secondary to the immune reconstitution.

## Conclusions

Congenital athymia is characterized by profound immunodeficiency due to the absence of a functioning thymus. The two primary manifestations of this disease are a result of the inability to produce immunocompetent T cells, leading both to immunodeficiency characterized by increased susceptibility to infection, and extrathymic T cell production leading to autologous GVHD. These patients can also present with additional symptoms related to associated genetic or syndromic conditions (FOXN1 deficiency, 22q11.2 deletion, CHARGE, and cDGS). Despite the currently available literature on congenital athymia, gaps remain, particularly in the full understanding of the etiology of congenital athymia. Future work should focus on improving the clinical management and treatment options available to patients with congenital athymia.

## Data Availability

Not applicable.
